# Vasoactive Intestinal Peptide Deficiency Is Associated With Altered Gut Microbiota Communities in Male and Female C57BL/6 Mice

**DOI:** 10.3389/fmicb.2019.02689

**Published:** 2019-12-02

**Authors:** Manpreet Bains, Caleb Laney, Annie E. Wolfe, Megan Orr, James A. Waschek, Aaron C. Ericsson, Glenn P. Dorsam

**Affiliations:** ^1^Department of Microbiological Sciences, College of Agriculture, Food Systems and Natural Resources, North Dakota State University, Fargo, ND, United States; ^2^Metagenomics Center, Department of Veterinary Pathobiology, College of Veterinary Medicine, University of Missouri, Columbia, MO, United States; ^3^Department of Statistics, College of Science and Math, North Dakota State University, Fargo, ND, United States; ^4^Intellectual and Developmental Disabilities Research Center, Department of Psychiatry and Biobehavioral Sciences, Semel Institute for Neuroscience and Human Behavior/Neuropsychiatric Institute, University of California, Los Angeles, Los Angeles, CA, United States

**Keywords:** microbiome, neuropeptide, gastrointesinal tract, intestinal epithelial cells, intestinal epithelial barrier, obesity, inflammatory bowel disease, Crohn’s disease

## Abstract

Vasoactive intestinal peptide (VIP) is crucial for gastrointestinal tract (GIT) health. VIP sustains GIT homeostasis through maintenance of the intestinal epithelial barrier and acts as a potent anti-inflammatory mediator that contributes to gut bacterial tolerance. Based on these biological functions by VIP, we hypothesized that its deficiency would alter gut microbial ecology. To this end, fecal samples from male and female VIP^+/+^, VIP^+/–^, and VIP^–/–^ littermates (*n* = 47) were collected and 16S rRNA sequencing was conducted. Our data revealed significant changes in bacterial composition, biodiversity, and weight loss from VIP^–/–^ mice compared to VIP^+/+^ and VIP^+/–^ littermates, irrespective of sex. The gut bacteria compositional changes observed in VIP^–/–^ mice was consistent with gut microbial structure changes reported for certain inflammatory and autoimmune disorders. Moreover, predicted functional changes by PICRUSt software suggested an energy surplus within the altered microbiota from VIP^–/–^ mice. These data support that VIP plays an important role in maintaining microbiota balance, biodiversity, and GIT function, and its genetic removal results in significant gut microbiota restructuring and weight loss.

## Introduction

Vasoactive intestinal peptide (VIP) is a 28 amino acid (AA) molecule. It was originally isolated from swine intestines and found to be vasoactive by dilating arterioles, thereby substantiating its name (Said and Mutt, 1970). VIP was later discovered in the central nervous system, which redirected the field to investigate its role as a neurotransmitter (Said and Rosenberg, 1976). These studies led to the understanding that VIP acts widely as a neurotransmitter/neuromodulator in the central and peripheral nervous systems. Moreover, VIP is a master circadian regulator, as its deletion in mice causes a cycling shift in wake/sleep duration with reduced food intake and body weights (Colwell et al., 2003; Vu et al., 2015). VIP is also delivered to immune organs by the peripheral nervous system, including the mucosa-associated lymphoid tissues of the gastrointestinal tract (GIT), where it plays a vital role in maintaining homeostasis (Lelievre et al., 2007).

Within the GIT, VIP, secreted from submucosal and myenteric neuron sources, regulates gastric acid secretion by the stomach, water/ion absorption in the large intestine, peristalsis by inhibiting smooth muscle contraction, and mucus secretion by Goblet cells (Lelievre et al., 2007). VIP maintains normal barrier function by promoting differentiation, proliferation, and cell adhesion of intestinal epithelial cells (IECs) (Wu et al., 2015). Metabolically, VIP-deficient mice have at least six dysregulated hormones, including glucagon and leptin (Martin et al., 2010). Within the lamina propria of the GIT, VIP blocks inflammation by downregulating co-stimulatory molecules on dendritic cells and generating tolerogenic FoxP3+ Tregs (Toscano et al., 2010). In some studies, VIP knockout mice were more susceptible to chemically-induced intestinal inflammation, and human inflammatory bowel disease (IBD) patients show a reduction in VIP positive nerves proximal to severe mucosal damage (Kimura et al., 1994; Wu et al., 2015). Lastly, VIP is modestly antimicrobial toward certain commensal bacteria (El Karim et al., 2008). In total, VIP is critical for GIT homeostasis and metabolism, and provides an anti-inflammatory “tone,” thereby contributing to immunological tolerance against gut microbiota.

The primary function of the GIT is nutrient absorption (Rowland et al., 2018). Humans and rodents utilize a collaborative strategy in harnessing energy from foods through a mutualistic relationship with their gut microbiota (Qin et al., 2010). The genes that the gut microbiota possess, called the microbiome, encode for enzymes that are critical for unlocking caloric energy from non-digestible polysaccharides in the form of short chain fatty acids (SCFA) (Ley et al., 2008; Tahara et al., 2018). “Germ-free” (GF) mice not possessing a gut microbiota weigh less, eat more, move more, store less fat, are resistant to diet-induced obesity, have an underdeveloped immune system, and are relatively resistant to chemically induced colitis (Backhed et al., 2007). These data highlight important influences by the gut microbiota on energy regulation, host metabolism, and immune development.

There is a paucity of research delineating the effects of VIP signaling on microbiota ecology despite the biological interactions between VIP and the gut microbiota. One study demonstrated that the administration of VIP into piglets caused gut bacterial compositional changes, and prevented diarrhea and weight loss in a bacterial infection model (Xu et al., 2014). The ratio between the two most common gut bacteria phyla, *Firmicutes* (F) and *Bacteroidetes* (B), was elevated by VIP, thereby providing evidence for its influence in shaping gut bacterial structure. We hypothesize that VIP deficiency will alter the gut microbiota ecology compared to WT littermates.

We report that genetic deletion of the VIP locus (VIP^–/–^, referred to as KO) resulted in substantial gut microbiota compositional changes with reduced F/B ratios, altered biodiversity, and decreased body weight compared to VIP^+/+^ (WT) and VIP^+/–^ (HET) littermates (*n* = 47). Importantly, male and female mice showed similar changes with certain exceptions. Predictive analysis by Phylogenetic Investigation of Communities by Reconstruction of Unobserved States (PICRUSt) showed changes predictive of an increase in anabolic metabolism and a decline in sugar and fiber uptake. In summary, these data support that the VIP neuropeptide plays an important role in mouse gut homeostasis, and its deficiency is associated with changes in microbiota structure, biodiversity, and decreased body weight.

## Materials and Methods

### Mice

**FIGURE 1:**
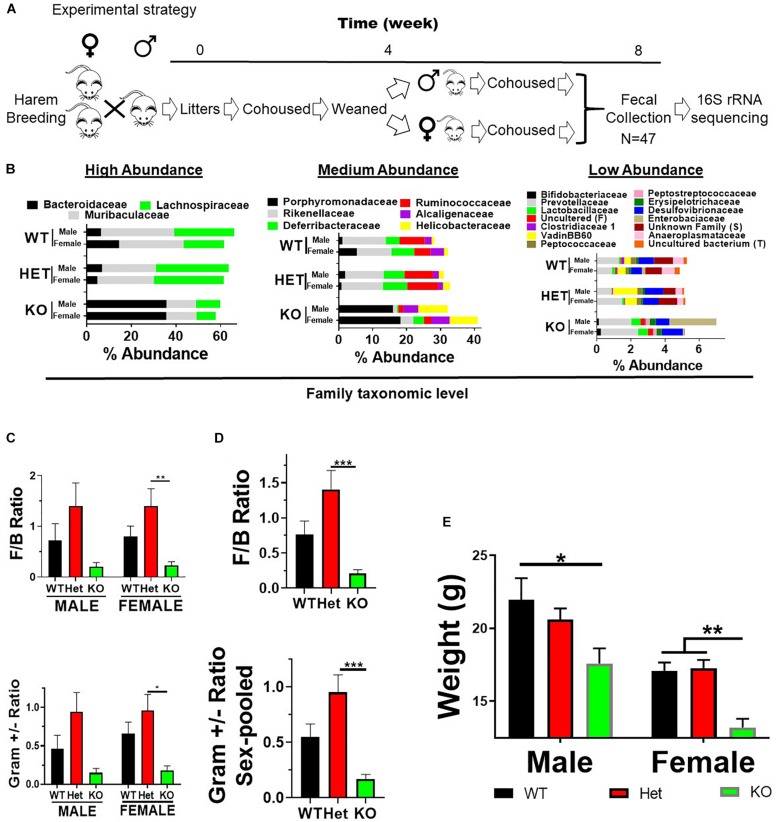
VIP deficiency results in fecal microbiota compositional changes and weight loss. **(A)** Horizontal line represents time in weeks as indicated. Breeding cages were established (week 0) and subsequent litters co-housed with parents until weaned by sex (4 weeks). Pups were housed for an additional 4 weeks following fecal collection on Thursday/Friday (week 8) beginning at 10 AM. Fecal samples were used for DNA extractions followed by 16S rRNA sequencing. **(B)** Horizontal stacked-bar graphs with each color representing % abundance means corresponding to the family name in the same color above the graph. Graphs are organized into high- (left panel), medium- (middle panel), and low-% abundance (right panel) family members with genotype and sex as indicated. **(C,D)** Bar graphs based on % abundances representing **(C)** *Firmicutes* to *Bacteroidetes* (F:B) phyla ratios ± SEM or **(D)** Gram-positive to Gram-negative ratios ± SEM from male and female (left panel), or sex-pooled (right panel) samples. **(E)** Bar graph representing mean body weights ± SEM grouped by sex and genotype as indicated (*n* = 25/genotype). ^∗^*p*-value ≤ 0.05, ^∗∗^*p*-value ≤ 0.01, ^∗∗∗^*p*-value ≤ 0.001.

Vasoactive intestinal peptide heterozygous (HET) breeders were a kind gift from Professor James Waschek at the University of California, Los Angeles (Colwell et al., 2003). Mice were bred at North Dakota State University, and research was approved by the NDSU Institutional Animal Care and Use Committee. Mice were housed in polycarbonate cages containing Alpha-Dri paper bedding (Animal Care Systems, Centennial, CO, United States), with access to 5001 LabDiet mouse chow (St. Louis, MO, United States) and tap water *ad libitum* with a 12 h light/dark cycle. The 5001 LabDiet ingredients can be found at http://www.youngli.com.tw/ezfiles/youngli/img/img/12858/5001.pdf. Briefly, it contains 24.1% protein, 11.4% fat, 5.2% fiber, 6.9% ash, and nitrogen-free extracts 48.7%. Mice were ear clipped for identification and tail biopsies collected for PCR genotyping (Colwell et al., 2003). VIP HET breeding cages (1 male and 2 females) with subsequent litters were cohoused with parents until weaned at 4 weeks of age. Male or female pups from weaned liters were cohoused for an additional 4 weeks following fecal collection on Thursday/Friday (week 8) beginning at 10 AM (Figure 1A). Fecal samples were used for DNA extractions followed by 16S rRNA sequencing.

### Mouse Genotyping

DNA was extracted from tail biopsies using Sigma–Aldrich extraction kit (St. Louis, MO, United States). Briefly, PCR reactions were performed with a unique 5′-wild type, a unique 5′-mutant and a common wild-type 3′-primer as published (Colwell et al., 2003). PCR reactions (20 μl) containing 1× GoTaq^®^ G2 master mix (Promega, Madison, WI, United States), primers (312.5 nM), and 2 μl of DNA template (1/20 dilution in TE) or nuclease-free water were amplified by PCR with the following parameters: 94°C(3:00) + [94°C(0:15), 62°C (0:45), 72°C(0:45)] × 40. Reactions were separated by agarose gel electrophoresis, visualized by UV light (254 nm) using EZ-Vision dye (Amresco, Radnor, PA, United States), and photographed with a digital camera (Alpha Innotech). VIP HET breeders yielded pups with the expected Mendelian frequency of 1:2:1 for WT:HET:KO, respectively.

### Fecal Collection

Mice were placed into autoclaved-sterile cages without bedding and allowed to defecate normally as published (Ericsson et al., 2018). Briefly, two freshly evacuated fecal pellets were collected per mouse using sterile toothpicks and placed into sterile 2 ml polypropylene microcentrifuge tubes. Samples were immediately frozen on dry ice and shipped overnight to the University of Missouri for 16S rRNA sequencing.

### DNA Extraction

Following mechanical disruption using a TissueLyser II from Qiagen (Venlo, Netherlands), fecal DNA was precipitated from samples, resuspended, and then re-extracted (purified) using the DNeasy Blood and Tissue kit and the DNeasy kit (Qiagen, Venlo, Netherlands), an approach adapted from Yu and Morrison (2004) and published previously (Ericsson et al., 2018).

### 16S rRNA Library Preparation and Sequencing

Bacterial 16S rRNA amplicons were constructed via amplification of the V4 hypervariable region of the 16S rDNA gene with universal primers (U515F/806R), flanked by Illumina standard adapter sequences as published (Ericsson et al., 2018). Primers with unique sequence tags were used. Briefly, PCR reactions (50 μl) contained 100 ng of genomic DNA, forward and reverse primers (0.2 μM each), dNTPs (200 μM each), and Phusion High-Fidelity DNA Polymerase (1 U). PCR amplification was performed as follows: 98°C(3:00) + [98°C(0:15) + 50°C(0:30) + 72°C (0:30)] × 25 cycles + 72°C(7:00). Amplified products were purified by Axygen AxyPrep MagPCR Clean-up beads followed by magnetic purification. The final amplicon pool was evaluated using the Advanced Analytical Fragment Analyzer automated electrophoresis system, quantified with the Qubit fluorometer using the quant-iT BR dsDNA reagent kit, and diluted according to Illumina’s standard protocol for sequencing by MiSeq.

### DNA Data Analysis

Assembly, binning, and annotation of DNA sequences were performed as published (Ericsson et al., 2018). Briefly, contiguous DNA sequences were assembled using FLASH software, and culled. Qiime v1.9 software was used to perform *de novo* and reference-based chimera detection and removal, and contiguous sequences assigned to operational taxonomic units (OTUs) via *de novo* OTU clustering and a criterion of 97% nucleotide identity (Ericsson et al., 2018). Taxonomy was assigned to selected OTUs using BLAST against the SILVA database (release 132) of 16S rRNA sequences and taxonomy. Principal coordinate analysis (PCoA) was performed using ¼ root-transformed OTU relative abundance data, and alpha (α)-diversity indices were determined using the Past 3.15 software package (Hammer and Harper, 2011). The University of Missouri Informatics Research Core Facility predicted the functional capacity of fecal samples from VIP KO mice by using the PICRUSt software package in an effort to predict metabolic pathways present at different levels between genotype (Langille et al., 2013).

### Statistics

Differences between groups in richness and α-diversity metrics were tested using a one-way analysis of variance (ANOVA) performed via a general linear model in SigmaPlot 13.0. Differences between groups in the relative abundance of 25 independently filtered OTUs with the highest loading scores were tested using a similar method although data were ¼-root transformed to normalize for high sparsity. Differences between genotype in beta (β)-diversity were determined via one-way permutational multivariate analysis of variance (PERMANOVA) of Euclidian distances using Past 3.15 (Hammer and Harper, 2011). When making comparisons among multiple taxonomic groups, ANOVA was performed by Graphpad using Bonferroni’s method to correct for multiple testing. For volcano plots, one-way ANOVA was performed to compare the genera’s mean (transformed) relative abundances (%) based on genotype comparisons (HET vs. WT, KO vs. WT, KO vs. HET) to calculate *p*-values. These *p*-values from all taxonomic groups and all comparisons were adjusted using the Bejamini and Hochberg approach (Benjamini and Hochberg, 1995). The negative log_2_ of these *p*-values were plotted against their corresponding sample fold changes for males and females.

### Data Availability Statement

The raw data supporting the conclusions of this manuscript will be made available by the authors, without undue reservation, to any qualified researcher.

## Results

### VIP Deficiency Results in Fecal Microbiota Compositional Changes in Male and Female Mice

Vasoactive intestinal peptide plays a fundamental homeostatic role in the GIT (Lelievre et al., 2007; Wu et al., 2015). Based on this activity, we hypothesized that VIP deficiency would alter gut microbial ecology. To this end, fecal samples from male and female WT (*n* = 17), HET (*n* = 16), and KO (*n* = 14; *n* = 47 total) littermates were collected, and 16S rRNA sequencing conducted using the Illumina platform (Figure 1A). A total of 3,582,783 high-quality rRNA 16S sequences were generated with an average of 76,229 sequences per sample. One male KO sample did not reach a significant number of DNA reads and was removed from the data set. This dataset consisted of 827 OTUs, which annotated to a total of 59 taxonomies. DNA reads from all fecal samples identified 10 phyla, with the 4 most abundant phyla for male, female, and total Taxa per phylum were: *Bacteroidetes* (59.88%, 54.26%; 11 Taxa) > *Firmicutes* (24.02%, 35.46%; 33 Taxa) >> *Deferribacteres* (7.08%, 4.15%, 1 taxon) = *Proteobacteria* (3.76%, 6.07%; 6 Taxa), representing 97.0–99.4% relative OTU abundance (referred to as % abundance). These four most abundant phyla represented 51 total Taxa. By two-way ANOVA, 7 of these 10 phyla reached statistical significance for genotype, but no statistical differences were observed between sexes (Table 1). Stacked bar graphs representing means of high and low % abundances for the seven statistically significant phyla, class, order, and genus are illustrated (Supplementary Figures S1A–D). These seven phyla represented 12 classes, 13 orders, 23 families, and 59 genera, and are represented at the family level (Figure 1B). Analyses between sex-pooled WT/HET versus KO, WT versus KO, WT versus HET, and HET versus KO revealed 28 (10 enriched, 18 depleted), 5 (2 enriched, 3 depleted), 5 (5 enriched, 0 depleted), and 9 (1 enriched, 8 depleted) genera that showed statistical significance (*p*-value ≤ 0.05). Figures 1C–D (top panels) illustrate the *Firmicutes* to *Bacteroidetes* (F/B) ratio for each genotype, with male and female WT and HET mice showing higher ratios compared to KO mice, but unlike HETs, WT mice did not reach statistical significance either separately or when sex-pooled. Similar profiles were observed when Gram-positive (*Actinobacteria* and *Firmicutes*) to Gram-negative (*Bacteroidetes*, *Deferribacteres*, and *Proteobacteria*) ratios were graphed (Figures 1C–D) (bottom panels). Lastly, statistically significant weight loss was seen in both male and female KO mice compared to WT and/or HET mice (Figure 1E). In total, male and female KO mice, and to a lesser extent HET mice, had radically altered gut microbial structures compared to WT mice, supporting its role in maintaining a stable gastrointestinal environment that influences the composition of the gut microbiota.

**TABLE 1:** List of detectable phyla categorized based on % abundance with ANOVA results.

**Abundance**	**Phyla**	**Two-way ANOVA**		
		**Genotype**	**Interaction**	**Sex**
High	*Bacteroidetes*	*P* = 0.0091	N.S.	N.S.
	*Firmicutes*	*P* = 0.001	N.S.	N.S.
Medium	*Deferribacteres*	*P* = 0.0170	N.S.	N.S.
	*Proteobacteria*	*P* = 0.0001	N.S.	N.S.
Low	*Saccharibacteria*	*P* = 0.0001	N.S.	N.S.
	*Tenericutes*	*P* = 0.0082	N.S.	N.S.
	*Verrucomicrobia*	N.S.	N.S.	N.S.
	*Chlamydiae*	N.S.	N.S.	N.S.
	*Cyanobacteria*	N.S.	N.S.	N.S.
	*Actinobacteria*	*P* = 0.0117	N.S.	N.S.

*N.S., not significant.*

### VIP Deficiency Causes Significant Changes in Biodiversity

**FIGURE 2:**
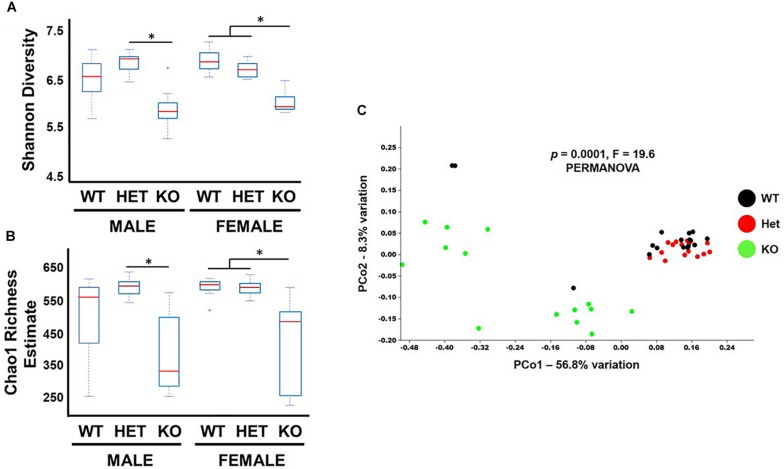
Biodiversity is reduced in KO mice. Box and whiskers plot illustrating α-diversity metrics from fecal samples as indicated by **(A)** Shannon and **(B)** Chao-1 indices. **(C)** PCoA graph of Unweighted Unifrac distances representing β-diversity for fecal samples from littermates as indicated. ^∗^*p*-value ≤ 0.05.

Bacterial diversity is an important variable thought to be directly related to the health of the gut microbiota (Morgan et al., 2013). Shannon diversity and Chao1 richness metrics are statistical calculations that can graphically represent intra-diversity (α-diversity) changes by using OTU read count data (Jin et al., 2015). These analyses indicated that samples from KO mice had lower α-diversity indices compared to WT and HET littermates (Figures 2A,B), although the comparison between male WT and KO samples did not reach statistical significance. To graphically represent inter-sample diversity (β-diversity), PCoA plots were used to compare compositional similarities and differences based on clustering and separation of samples, respectively (Jin et al., 2015). Figure 2C shows male and female KO samples clustering differently along the PC1 ordinate compared to WT and HET samples (PERMANOVA, *P* = 0.0001, F-19.6). Taken together, these results support the conclusion that the overall biodiversity of KO samples have reduced α-diversity and different β-diversity compared to HET and WT samples, irrespective of sex.

### VIP Deficiency Causes Phylogenetic Lineage Alterations Within the Gut Microbiota

**FIGURE 3:**
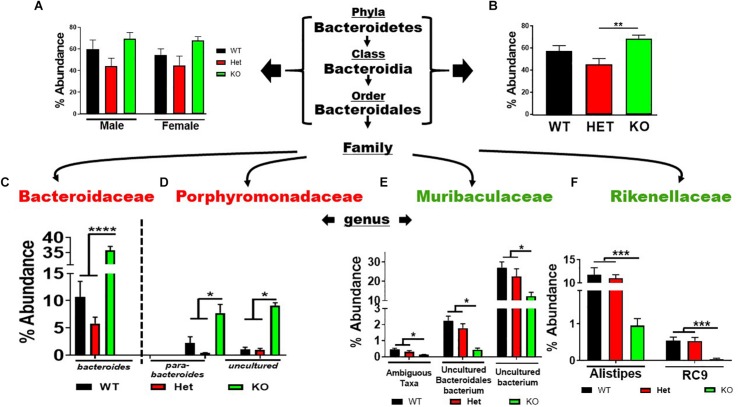
Net enrichment and reshuffling of genera in *Bacteroidetes* → *Bacteroidales* lineage in KO mice. **(A,B)** Bar graphs showing % abundance means ± SEM from **(A)** male and female, or **(B)** sex-pooled fecal samples with corresponding taxonomic levels indicated by arrows. **(C–F)** Four families as indicated branch off revealing a total of eight genera as shown demonstrating **(C,D)** enrichments, or **(E,F)** depletions of % abundance means ± SEM. ^∗^*p*-value = 0.05, ^∗∗^*p*-value ≤ 0.01, ^∗∗∗^*p*-value ≤ 0.001, or ^****^p-value ≤ 0.0001.

Analysis of the seven phyla that showed statistically significant differences between genotype revealed several notable outcomes. Regarding the *Bacteroidetes* phylum, there was a similar trend in % abundances between male and female samples for the *Bacteroidetes* → *Bacteroidales* lineage, with HET samples showing the lowest % abundances compared to WT and KO mice, but not reaching statistical significance (Figure 3A). Due to their similar profiles, we combined male and female replicates to reveal a statistically significant net increase in % abundance between KO and HET samples, but WT mice did not reach statistical significance (Figure 3B). However, in lower taxonomic levels more consistent statistical changes between genotype were observed. Comparing KO to WT and HET mice, there were enrichments in *Bacteroides*, *Parabacteroides*, and an *Uncultured* genus of the Porphyromonadaceae family. Concomitantly, these enrichments were partially compensated by a depletion of three genera making up the Muribaculaceae family, and two genera of the Rikenellaceae family (Figures 3C–F), with similar % abundance profiles between male and female samples (Supplementary Figures S2A–D). We conclude that VIP deficiency is associated with a net enrichment in % abundances within the *Bacteroidales* order compared to WT and HET mice. Perhaps more importantly, there was a substantial reshuffling of eight genera with blooms in the Bacteroidaceae and Porphyromonadaceae families but depletions in Muribaculaceae and Rikenellaceae families in KO compared to both WT and HET mice.

**FIGURE 4:**
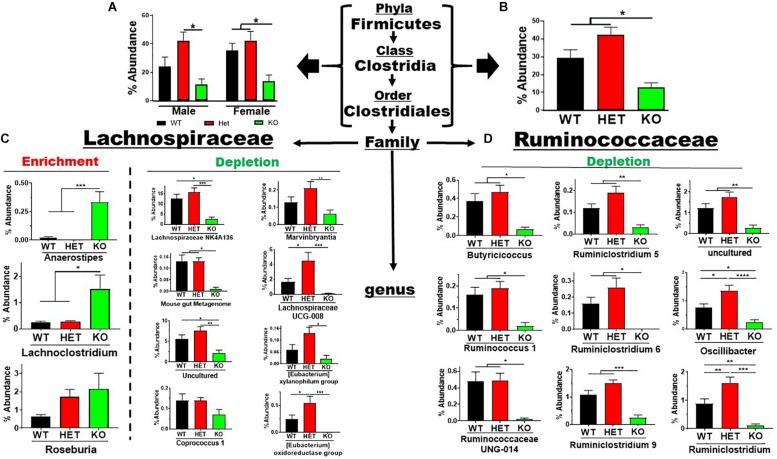
Net depletion in *Firmicutes* → *Clostridiales* lineage in KO mice. **(A)** Bar graphs showing % abundance means ± SEM from **(A)** male and female, or **(B)** sex-pooled fecal samples with corresponding taxonomic levels indicated by arrows. **(C,D)** The two most abundant *Firmicutes* families totaling 20 genera with means ± SEM of % abundance changes organized based on an enrichment or depletion in KO mice from **(C)** Lachnospiraceae, or **(D)** Ruminococcaceae lineages. ^∗^*p*-value ≤ 0.05, ^∗∗^*p*-value ≤ 0.01, ^∗∗∗^*p*-value ≤ 0.001, or ^****^p-value ≤ 0.0001.

The Firmicutes phylum branched into the Bacilli → *Lactobacillales* and Clostridia → *Clostridiales* lineages representing 7 families and 26 genera. The low-abundant Lactobacillales order showed an enrichment in sex-pooled KO mice compared to HET and WT samples, which was maintained through the family and genus levels (Supplementary Figure S3A). Similar % abundance profiles were seen in male and female mice when analyzed separately (Supplementary Figures S3B,C). In contrast, the more abundant Clostridiales order revealed depletions in KO compared to WT and/or HET mice, irrespective of sex (Figure 4A). This order represented 5 families and 23 genera. Three (Colwell et al., 2003) low-abundant families representing four genera are graphed in Supplementary Figures S4A–C. Three (Colwell et al., 2003) of the four genera showed statistically significant % abundance changes due to genotype when samples were sex-pooled, as similar % abundance profiles were observed for male and female samples. The two remaining, high-abundant families were Lachnospiraceae and Ruminococcaceae, accounting for 30 and 42% total reads for WT and HET compared to only 13% for KO when pooling male and female samples. There were % abundance depletions from sex-pooled KO compared to WT and/or HET samples from 17 of the 20 genera (Figures 4B–D), most of which reached statistical significance. The remaining three genera showed enrichments in sex-pooled KO compared to WT and HET mice, with two genera reaching statistical significance. We also analyzed male and female Lachnospiraceae and Ruminococcaceae family data separately and discovered 11 had similar % abundance profiles. The remaining 9 genera displayed a lack of statistical significance between male WT and KO mice, which singularly made the % abundance profiles between sexes different (Supplementary Figure S5, red arrows). Moreover, these % abundances mirrored male KO mice, suggesting a possible influence. In total, 19 out of 26 genera resulted in % abundance depletions in KO compared to male and female WT and/or HET mice. The one caveat was that male WT mice displayed aberrant % abundancies in nearly 50% of the genera potentially caused by co-housing conditions and/or epigenetic changes.

**FIGURE 5:**
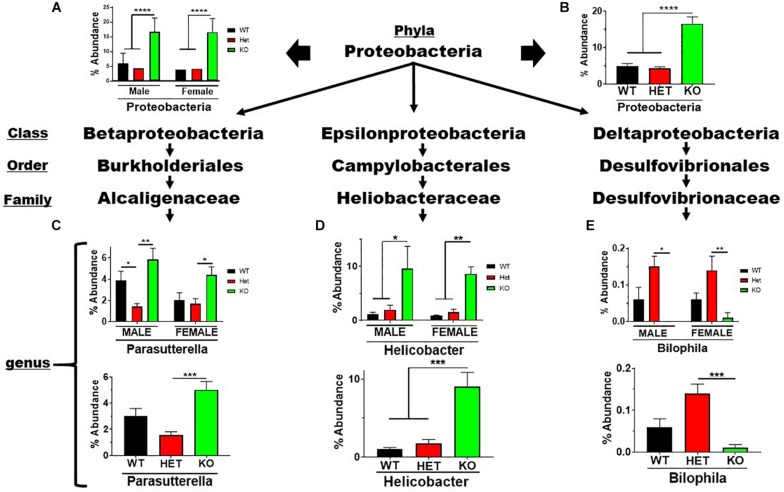
Net enrichment in the *Proteobacteria* phylum in KO mice. Bar graphs showing % abundance means ± SEM from **(A)** male and female, or **(B)** sex-pooled samples with corresponding taxonomic levels indicated by arrows. **(C–E)** Bar graphs showing means ± SEM from male and female **(top)**, or sex-pooled **(bottom)** samples from **(C)** *Beta*-, **(D)** *Epsilon*-, or **(E)** *Delta-proteobacteria* lineages. ^∗^*p*-value ≤ 0.05, ^∗∗^*p*-value ≤ 0.01, or ^∗∗∗^*p*-value ≤ 0.001, ^****^p-value ≤ 0.0001.

The Proteobacteria phylum contains pathobionts that can cause disease (Lee et al., 2017). This phylum presented an enrichment in % abundance from KO compared to WT and HET samples, irrespective of sex (Figures 5A,B). Betaproteobacteria and Epsilonproteobacteria orders representing two genera, *Parasutterella* and *Helicobacter*, displayed similar profiles observed at the phyla level (Figures 5C,D). In contrast, the low-abundance *Bilophila* genus in the *Deltaproteobacteria* order was significantly depleted in male and female KO compared to HET and WT samples (Figure 5E). These data support the notion that VIP signaling can alter Proteobacteria composition, and limit potentially pathogenic, Gram-negative bacteria in the *Parasutterella* and *Helicobacter* genera.

The remaining four phyla: *Actinobacteria*, *Deferribacteres*, *Saccharibacteria*, and *Tenericutes*, comprised a total of four genera, three of which were depleted, and one enriched, in male and female KO compared to HET and WT mice (Supplementary Figure S6). *Bifidobacterium*, *Mucispirillum*, *Candidatus Saccharimonas*, and *Anaeroplasma* genera reached statistical significance between KO and/or WT/HET sex-pooled samples, with similar profiles between male and female mice.

**FIGURE 6:**
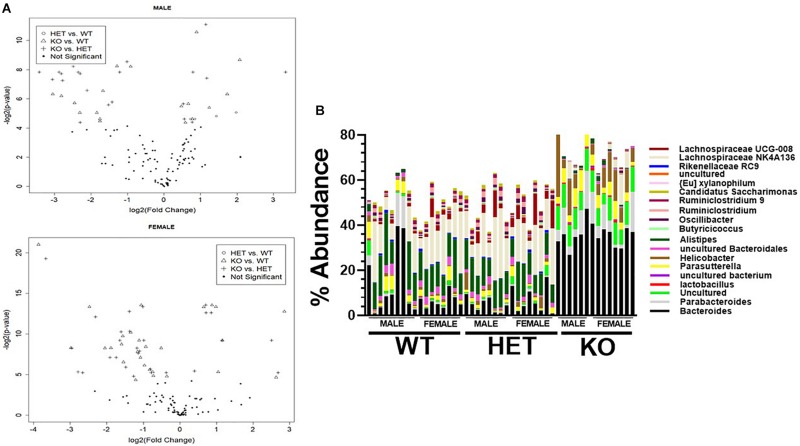
Identification of genera biomarkers due to VIP deficiency. **(A)** Volcano plots for male **(top)** or female **(bottom)** samples for indicated genotype comparisons. Not significant represents adjusted *p*-values ≥ 0.05. **(B)** A vertical stacked-bar graph with each color representing % abundance levels corresponding to the genus name of the same color at right for male and female samples from the indicated genotypes (*n* = 47).

We next attempted to identify the most relevant genera “biomarkers” based on fold-changes and adjusted *p*-values by generating volcano plots (Figure 6A). This analysis revealed, as stated above, that VIP deficiency caused both enrichments and depletions when compared to WT and HET samples (male: 9 and 8, female: 21 and 7; male: 14 and 7, female: 21 and 7). By cross-referencing genera changes that were common among sexes, a shorter list of 19 genera was identified that were consistently altered in KO versus WT and/or HET samples (Figure 6B and Table 2). More than half of these genera (El Karim et al., 2008) represented the Firmicutes phylum, of which eight were depleted supporting that this phylum is normally bolstered by VIP signaling. This overall depletion in *Firmicutes* genera was at the expense of blooms in the *Proteobacteria* and *Bacteroidetes* phyla representing more than half of the DNA reads. The *Bacteroidetes* phylum had an equal number of genera changes, with three blooming and three becoming depleted, supporting a redistribution of genera within this phylum due to VIP deficiency. We propose that these genera changes represent important biomarkers due to the loss of the GIT homeostatic and anti-inflammatory VIP peptide.

**TABLE 2:** Consistently altered genera from male and female fecal samples.

		**Fold-change**		**Adjusted *P*-value**	
**Phylum**	**Genus**	**Male**	**Female**	**Male**	**Female**
**Enriched**					
*Bacteroidetes*	*Bacteroides*	1.392	1.566	0.022	9.74*E*−05
	*Parabacteroides*	1.503	2.232	0.048	0.002
	*Uncultured*	1.858	1.976	0.001	9.52*E*−05
*Firmicutes*	*Lactobacillus*	1.713	2.061	0.041	0.025
	*Uncultured bacterium*	3.321	7.237	0.012	1.42*E*−04
*Proteobacteria*	*Parasutterella*	1.441	1.32	0.02	0.023
	*Helicobacter*	1.587	1.839	0.02	8.21*E*−05
**Depleted**					
*Bacteroidetes*	*Uncultured Bacteroidales*	0.409	0.7	0.003	0.003
	*Alistipes*	0.495	0.496	0.003	9.52*E*−05
	*Rikenellaceae RC9*	0.142	0.271	0.014	0.003
*Firmicutes*	*Lachnospiraceae NK4A136*	0.230	0.454	0.01	0.005
	*Lachnospiraceae UCG-008*	0.372	0.383	0.018	1.42*E*−04
	*EU Xylanphilum*	0.203	0.158	0.048	0.026
	*Uncultured*	0.183	0.331	0.019	0.002
	*Butyricicoccus*	0.202	0.564	0.03	0.02
	*Oscillibacter*	0.296	0.473	0.041	0.007
	*Ruminiclostridium*	0.296	0.339	0.045	0.003
	*Ruminiclostridium 9*	0.279	0.523	0.03	0.003
*Saccharibacteria*	*Candidatus Saccharimonas*	0.121	0.069	0.012	4.67*E*−07

### A Predicted Energy Surplus Within the Gut Microbial Community Due to VIP Deficiency

We employed the PICRUSt software as a predictive tool of the overall metagenomic capacity of these different microbial communities. Using this well-validated software, we predicted 33 enriched and 10 depleted functional modules in sex-pooled KO mice compared to both HET and WT samples (data not shown) (Langille et al., 2013). The KO microbiome had enhanced biosynthetic potential by elevating genes involved in gluconeogenesis and the pentose phosphate pathway leading to the biosynthesis of LPS, purines, vitamins, and amino acids (Table 3). These changes were coupled with differential metabolite transporter expression including ATP transporters bringing in sulfate, arginine, and glutamate, while excluding sugars and fiber. We conclude that VIP deficiency impacts predicted changes in the microbiota’s metabolism network by promoting: (1) carbon metabolism favoring the production of NAD(P)H, (2) biosynthesis of cellular metabolites, including LPS, and (3) altered import rates of luminal macromolecules, like reduced polysaccharide intake that may lower SCFA production (Tahara et al., 2018). Such changes could be indicative of an energy-rich microbiota environment.

**TABLE 3:** Predicted metabolic changes in VIP KO versus WT/HET fecal microbiota.

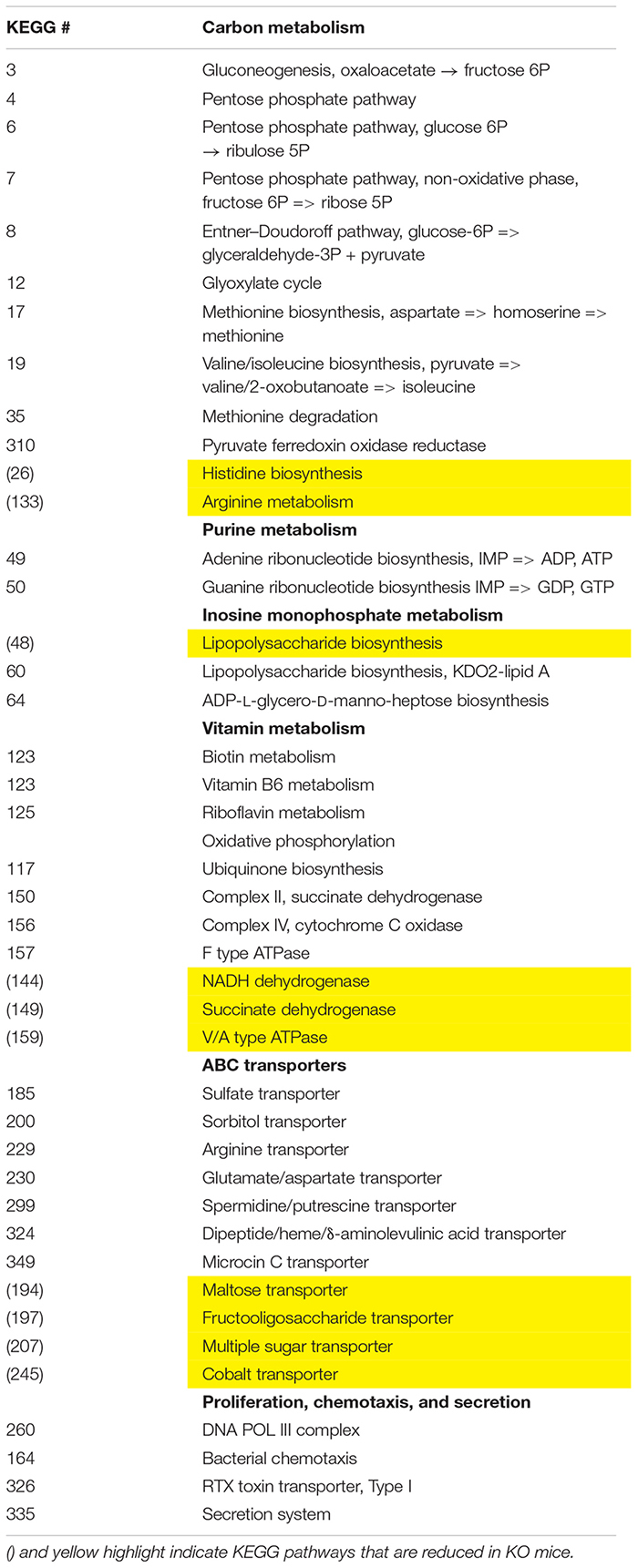

## Discussion

This study clearly demonstrates the importance of VIP in the establishment and/or maintenance of the gut microbiota, as its absence is associated with massive gut bacterial restructuring, changes in biodiversity, and reduction in body weight. To our knowledge, this is the first reported evidence demonstrating the phenotype of gut bacterial compositional changes in VIP KO mice (El-Salhy et al., 2017).

The major taxonomic changes found in KO mice compared to WT and HET littermates can be summarized by: (1) a net depletion in the Gram-positive *Firmicutes* phylum, (2) net increases in the Gram-negative *Bacteroidetes* and *Proteobacteria* phyla, and (3) a reshuffling of families and genera structures within these phyla. These gut microbiota changes resemble gut bacterial alterations associated with inflammatory disorders. For example, the IBD disorder, Crohn’s disease (CD), has shown decreases in the butyrate-producing *Firmicutes* phylum, a reduction in α-diversity and an enrichment in the *Bacteroides* genus (Kho and Lal, 2018). Increases in *Bacteroides* contribute to elevated LPS biosynthesis, which has been shown to induce IBD, and several *Bacteroides* pathobionts have been reported to be causative for the onset of IBD (Bloom et al., 2011). Gronbach et al. (2014) in 2014 showed that GF mice become more susceptible to chemically induced IBD when colonized with Gram-negative bacteria. One mechanism by which *Bacteroides* could cause disease might be mediated by LPS-induced TLR4 signaling that favors Th17 effector cells and reduces tolerogenic Treg frequency. Moreover, VIP signaling decreases the expression of TLR4, while inducing the expression of anti-inflammatory TLR molecules (Jiang et al., 2012). Therefore, VIP deficiency resulting in elevated *Bacteroides* could trigger enhanced LPS→TLR4 signaling and contribute to a proinflammatory intestinal environment leading to a breakdown in commensal tolerance and susceptibility to inflammatory disorders like CD. Celiac (Nadal et al., 2007), type-I diabetes (Murri et al., 2013), and Lupus (Luo et al., 2018) all have elevations in *Bacteroidetes* and/or *Proteobacteria*. Lupus manifests a reduction in α-diversity and *Alistipes*, and Type I diabetic mice show a reduction in the F/B ratio with a depletion of *Firmicutes*. In contrast, Celiac disease showed a decrease in *Bifidobacterium* (Nadal et al., 2007) and type I diabetes had a reduction in *Actinobacteria* (Murri et al., 2013).

Decades of research have revealed VIP as a potent anti-inflammatory mediator (for review see Abad and Tan, 2018). Mechanisms controlling this immunomodulatory action for VIP can be summarized by the downregulation of co-stimulatory molecules on dendritic cells, and the induction of tolerogenic FoxP3^+^ Tregs (Toscano et al., 2010). Curiously, there remains a large degree of controversy with respect to VIP’s role in IBD, including CD (Abad and Tan, 2018). In human IBD, VIP plasma levels were reduced in mild cases, but elevated in severe cases, and was suggested as a diagnostic tool for IBD prognosis (Duffy et al., 1989). In the GIT, IBD patients had reduced, increased, or no change in VIP expression (El-Salhy et al., 2017). A possible explanation for differential changes in VIP levels is that its expression represents a vast number of possible cell types, including most epithelial cells, and cells of the nervous, endocrine, and immune systems (Dorsam et al., 2011). Consistent with the discordant data in human IBD, mouse inflammatory immune studies are also riddled with seemingly inconsistent results. Some studies reported that exogenously added VIP to human IBD mouse models had less severe histopathology scores and diminished inflammation (Abad and Tan, 2018). In stark contrast, researchers have shown exacerbating effects by VIP administration in chemically induced colitis IBD models showing more severe histopathology and elevated inflammatory cytokine levels (Abad et al., 2015). Using VIP KO mice, different studies showed an exacerbation or an amelioration of chemically induced colitis (Abad et al., 2015; Wu et al., 2015). The notion that VIP could have both pro- and anti-inflammatory actions is not unreasonable, as VIP differentially regulates IL-6 expression, which can act as a pro- and anti-inflammatory cytokine (Huang et al., 2012). Another explanation could be due to gut microbiota structure differences as recently suggested by Wu et al. (2015). Our present study clearly reveals substantial changes in the gut bacterial ecology that when combined with environmental factors could shift the balance between inflammation versus tolerance helping to explain the vastly different results observed in the VIP IBD literature. Indeed, significant changes in gut microbiota structure have been demonstrated due to different geographical locations of mouse vivariums (Reinoso Webb et al., 2018). These data emphasize the importance for researchers to declare environmental conditions, including mouse chow, bedding, and cage aeration specifics (Ericsson et al., 2018).

This study shows the opposite F:B ratio as observed in two different obese mouse models (Ley et al., 2005; Nishitsuji et al., 2017). Couple this difference with reduced body weights, defective fat storage, and elevated blood glucose, insulin, and leptin levels, and the VIP deficient mice appear to also have an anorexic, prediabetic phenotype (Vu et al., 2015). Vu et al. (2015) reported weight loss in male KO mice, and our present data confirm similar male weight loss and also confirm weight loss in female KO compared to WT and HET mice. In humans, the VIP signaling axis was found to be the strongest associated pathway linked to obesity development out of 963 analyzed by a genome wide analysis (Liu et al., 2010). Other human studies showed elevated plasma VIP levels in obese males and anorexic females, but reduced plasma levels in obese females, suggesting a possible sex difference in humans (Barreca et al., 1989; Baranowska et al., 2000). Fasting does alter gut microbiota structure and therefore it cannot be ruled out that reduced food intake by VIP KO mice could contribute to the observed changes in gut microbiota ecology (Li et al., 2017). Nonetheless, these data strengthen the connection between VIP signaling, the gut microbiota, and energy homeostasis.

Another neuropeptide that shares 68% amino acid homology to VIP, called pituitary adenylate cyclase activating polypeptide (PACAP), also manifests gut microbiota compositional changes when genetically deleted in mice (Miyata et al., 1989; Heimesaat et al., 2017). Gut microbiota structure changes in PACAP KO mice were evaluated by qPCR using primers to 16S genes from fecal samples over time. This study showed a modest enrichment for *Bacteroides* from PACAP KO mice, and there were enrichments in enterobacteria from the Proteobacteria phylum. In contrast to VIP deficiency-induced gut microbiota compositional changes, PACAP KO mice had no detectable depletions in the *Firmicutes* → *Clostridium* lineage, and *Bifidobacterium* was significantly decreased. This later observation was further speculated by the authors to be relevant to increased vulnerability to intestinal diseases (Heimesaat et al., 2017). One major reason for the differences in gut microbiota between PACAP and VIP KO mice is that the modes of action of these two peptides within the gut and elsewhere are different. PACAP is a 38 AA neuropeptide that possesses similar exon/intron organization with the VIP gene, and PACAP’s AA sequence has remained nearly unchanged for over 750 million years of evolution from tunicate to human (96% identical) (Nussdorfer and Malendowicz, 1998). PACAP and VIP bind with equal affinity to VIP/pituitary adenylate cyclase activating polypeptide (VPAC1) and VPAC2 receptors, while a third receptor, PAC1, binds to PACAP with high-affinity, and VIP with low-affinity (Vaudry et al., 2009). The distinct phenotypes in PACAP and VIP KO mice could be explained by differences in receptor binding preference and/or accessibility, and signal transduction pathways. PACAP deficient mice have other similar and different phenotypes to that of VIP KO mice. PACAP is widely expressed in the brain and is delivered to peripheral tissues, including the gut. Some of its main and unique biological actions are cytoprotective against cellular stressors, like ischemia/reperfusion damage, while similar to VIP, PACAP acts as an anti-inflammatory mediator (Reglodi et al., 2012). At baseline, PACAP KO mice did not show intestinal morphological changes as were observed with VIP deficient mice (Lelievre et al., 2007; Nemetz et al., 2008). During DSS-induced intestinal colitis, both VIP- and PACAP-deficient mice showed greater histopathology scores, with elevated proinflammatory cytokine expression. However, PACAP KO mice resulted in higher mortality rates and colorectal cancer development not seen in VIP KO mice (Azuma et al., 2008; Nemetz et al., 2008). Taken together, both PACAP and VIP play important roles in the gut as anti-inflammatory and cytoprotective meditators, and their loss results in gut microbiota community alterations. However, there were definite differences in gut microbiota suggesting that the modes of action between PACAP and VIP are distinct and not entirely overlapping. The observed differences in the gut microbiota structures between these two KO mouse strains might be explained by experimental methods employed by the PACAP study and our present VIP study. For example, it has been reported that microbiota composition comparisons between studies using different techniques (16S deep sequencing versus qPCR) can often lead to differences (Robinson et al., 2016). Additional research is clearly needed to further investigate how the VIP/PACAP signaling axis influences the composition and function of the gut microbiota.

Mechanisms influencing gut bacterial composition by VIP are likely multifactorial. In addition, to the aforementioned action on mucosal immunity, VIP signaling is crucial for maintaining GIT morphological architecture as KO mice have hyperplasia of smooth muscle within the muscularis plexus, causing their intestines to weigh more, but become shorter (Lelievre et al., 2007). VIP also provides survival, proliferative, and migratory signals for IECs, as KO mice showed elevated IEC apoptosis, coupled with reduced proliferative and migratory capacity (Wu et al., 2015). The same authors noticed a reduction in goblet cell number coupled with a decline in MUC2 synthesis and secretion, the major component of mucus, and a downregulation in caudal-related homeobox transcription factor 2, an intestine-specific protein known to regulate MUC2 expression and IEC homeostasis. Most of these phenotypes were rescued by exogenous addition of VIP ligand indicating that the observed effects were directly mediated by VIP (Wu et al., 2015). Decrease in MUC2 secretion due to VIP deficiency was also observed by an earlier study (Lelievre et al., 2007). Support for reduced luminal mucus driving gut bacterial compositional changes has been reported by Wu et al. (2018) showing that MUC2 deficiency leads to a depletion of the butyrate-producing *Firmicutes* → *Rumminococcaceae* clade. However, differences between MUC2 and VIP deficient mice were noted. MUC2 deficient mice possessed an “obesity-like” increase in the F:B ratio, depletion of two probiotics, *Lactobacilli* and *Lachnospiraceae*, enrichment in potential pathobionts, *Erysipelotrichaceae* and *Desulfovibrio* and elevated α-diversity, the latter being a common occurrence in MUC2 deficient mice with colorectal cancer development (Lu et al., 2016). Taken together, defective MUC2 expression and/or secretion cannot fully explain the altered gut microbiota compositional changes, thus emphasizing that the precise mechanism by which VIP deficiency causes changes in the mouse gut microbiota is presently unknown. More in depth mechanistic studies are required to delineate the extent to which a direct and/or indirect mechanism(s) controls gut microbiota structure changes due to VIP deficiency.

The present study does have some limitations. First, measurements aimed at evaluating the proinflammatory status for VIP KO mice were not conducted as several previous reports have documented proinflammatory cytokine increases in both lung and gut tissue, and systemically in blood (Abad and Tan, 2018). Moreover, the similar gut microbiota changes seen in the present study compared to inflammatory disorders like Crohn’s and Celiac disease further support a common inflammatory tone within the GITs of VIP KO mice. Importantly, this conclusion would remain valid regardless of additional data supporting or debunking a proinflammatory status in VIP KO mice. Having said this, it is a major future goal to investigate the extent of the proinflammatory status of VIP KO mice within the GIT by conducting a detailed immune-profiling study investigating changes in innate and adaptive immune subpopulations by flow cytometry, along with inflammatory cytokine measurements, antimicrobial peptide expression profiles, and IgA levels. Second, a reduction in food consumption by VIP KO mice could explain the microbiota changes from this study as diet is a powerful environmental factor in regulating the composition of the gut microbiota (Ley et al., 2005). One mitigating factor to a reduced-food consumption mechanism is that the same VIP KO mice eat about threefold more food during the 12 h light phase complicating the interpretation that these mice simply eat “less” food. Due to this mitigating factor to the feeding behavior of VIP KO mice, as well as the significant biological influences of VIP in the health of the GIT, we propose that a small average reduction in food consumption for VIP KO mice is unlikely to exclusively drive the observed phenotype of a disrupted microbiota. Fasting and/or antibiotic knockdown of the gut microbiota experiments might shed light on whether reduced food intake is a contributing factor. Lastly, investigations by qPCR for specific species within genera identified in this study was not conducted and thought to be beyond the scope of this report. However, with our present dataset, a comprehensive investigation can now be initiated to measure species within abundant genera, such as *Bacteroides* and *Helicobacter* that represented almost half of all Taxa identified.

In summary, VIP deficiency results in gut microbiota community changes, reduced numbers of sequenced Taxa, and leaner body weights. Future studies focusing on which VIP receptor mediates these changes are currently in progress. Moreover, additional research is necessary to better delineate the impact that VIP signaling has on the spatial location of bacteria along the GIT, IEC biology, immune pathways, and host energy homeostasis.

## Data Availability Statement

This manuscript contains previously unpublished data. The name of the repository and accession number(s) are not available.

## Ethics Statement

The animal study was reviewed and approved by the NDSU IACUC Committee.

## Author Contributions

MB and CL performed the mouse husbandry, genotyping, and fecal pellet collections. MB wrote the sections of the manuscript. MO calculated the adjusted *p*-values and fold-changes for the dataset and produced the volcano graphs, and wrote certain sections of the manuscript. JW provided the VIP knockout breeders and critically edited the manuscript. AE provided advise for the experimental design, provided graphical representation for some figures, and critically edited the manuscript. GD devised the experimental design, established collaborations with MO, JW, and AE, interpreted all the data, graphed the data, organized the tables, and wrote and edited the manuscript.

## Conflict of Interest

The authors declare that the research was conducted in the absence of any commercial or financial relationships that could be construed as a potential conflict of interest.

## Supplementary Material

FIGURE S1 VIP deficiency causes phylogenetic lineage alterations within fecal gut microbiota. Horizontal stacked-bar graphs for **(A)** phyla, **(B)** class, **(C)** order, and **(D)** genus taxonomic levels. Color represent means of % abundance levels corresponding to the taxonomic name for that same color associated with each graph. Graphs are organized into high-, medium, and low % abundance categorized by genotype and sex. Forty of the 59 genera are graphed.

FIGURE S2 Net enrichment and reshuffling of genera in *Bacteroidetes* → Bacteroidales lineage in KO mice. **(A–D)** Four families branch off revealing a total of eight genera represented as bar graphs with % abundance means ± SEM categorized by sex and genotype as indicated for **(A)** Bacteroidaceae, **(B)** Porphyromonadaceae, **(C)** Muribaculaceae, and **(D)** Rikenellaceae. ^∗^*p*-value ≤ 0.05, ^∗∗^*p*-value ≤ 0.01, or ^****^*p*-value ≤ 0.0001.

FIGURE S3 Depletions in Bacilli→ Lactobacillales lineage in KO mice. **(A)** Bar graphs showing % abundance means ± SEM for male versus female **(top)**, or sex-pooled **(bottom)** samples for indicated genotypes. **(B,C)** Bar graphs showing % abundance means ± SEM for male versus female **(top)**, or sex-pooled **(bottom)** samples for indicated taxonomic levels and genotypes. ^∗^*p*-value ≤ 0.05, ^∗∗^*p*-value ≤ 0.01 and ^∗∗∗^*p*-value ≤ 0.001.

FIGURE S4 Differential genera changes from three low abundant *Clostridiales* order in KO mice. Bar graphs showing % abundance means ± SEM for male versus female **(top)**, or sex-pooled **(bottom)** samples for indicated genotypes from **(A)** Peptostreptococcaceae, **(B)** *Clostridiales* VandinBB60, or **(C)** Peptococcaceae families. ^∗^*p*-value ≤ 0.05, ^∗∗^*p*-value ≤ 0.01 and ^∗∗∗^*p*-value ≤ 0.001.

FIGURE S5 Consistent sex differences between male and female WT mice. Bar graphs representing 9 of the 20 genera showing discordant data between male versus female samples (red arrow). Data are represented as bar graphs with % abundance means ± SEM. ^∗^*p*-value ≤ 0.05, ^∗∗^*p*-value ≤ 0.01, or ^∗∗∗^*p*-value ≤ 0.001.

FIGURE S6 Changes in the remaining four phyla lineages that showed statistical significance due to genotype. Taxonomic levels are indicated on the left. Bar graphs showing % abundance means ± SEM for male versus female **(top)**, or sex-pooled genotypes **(bottom)** as indicated for **(A)** *Actinobacteria* → *Bifidobacterium*, **(B)** *Deferribacteres* → *Mucispirillum*, **(C)** *Tenericutes* → *Anaeroplasma*, and **(D)** *Saccharibacteria* →*Candidatus Saccharimonas* lineages. ^∗^*p*-value ≤ 0.05, ^∗∗^*p*-value ≤ 0.01 and ^****^*p*-value ≤ 0.0001.
