# Supplementary figures and images for: Vasoactive Intestinal Peptide Deficiency Is Associated With Altered Gut Microbiota Communities in Male and Female C57BL/6 Mice
Source: Front Microbiol. 2019 Dec 2;10:2689. doi: 10.3389/fmicb.2019.02689 (PMC6900961; doi:10.3389/fmicb.2019.02689)

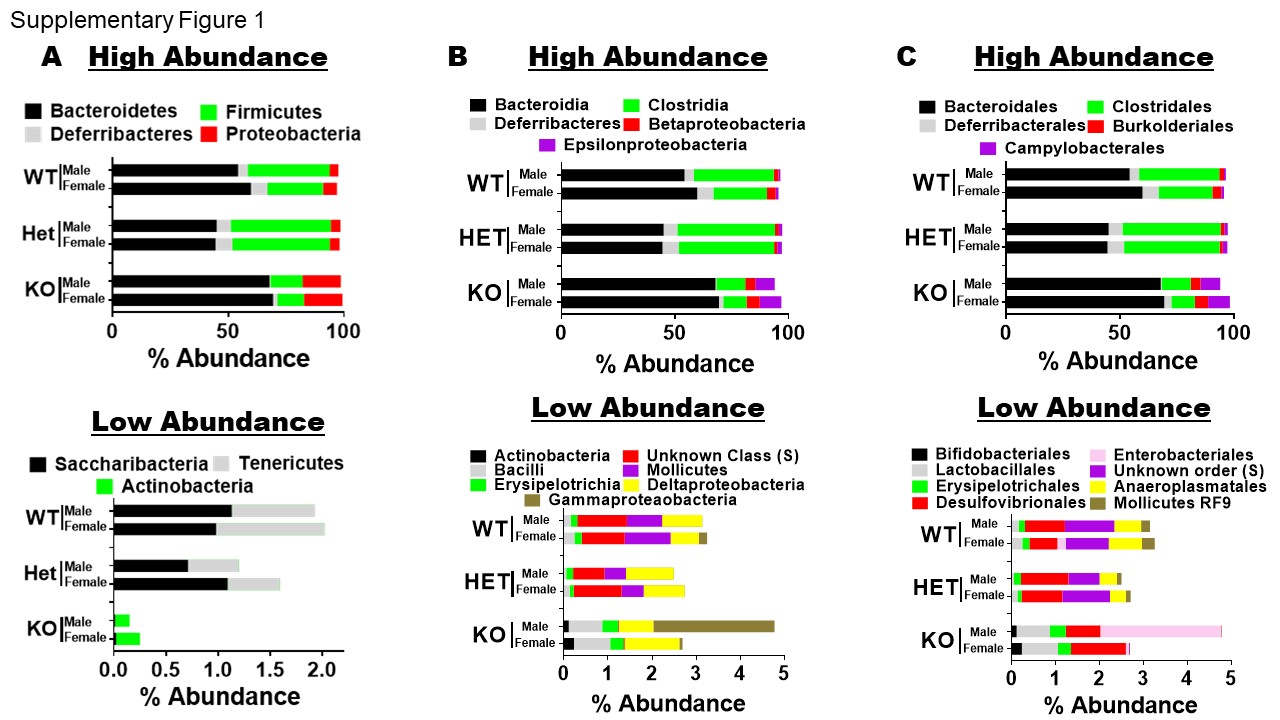

Supplement: FIGURE S1 — VIP deficiency causes phylogenetic lineage alterations within fecal gut microbiota. Horizontal stacked-bar graphs for (A) phyla, (B) class, (C) order, and (D) genus taxonomic levels. Color represent means of % abundance levels corresponding to the taxonomic name for that same color associated with each graph. Graphs are organized into high-, medium, and low % abundance categorized by genotype and sex. Forty of the 59 genera are graphed. [file Data_Sheet_1.zip › Image 1A-C.JPEG]

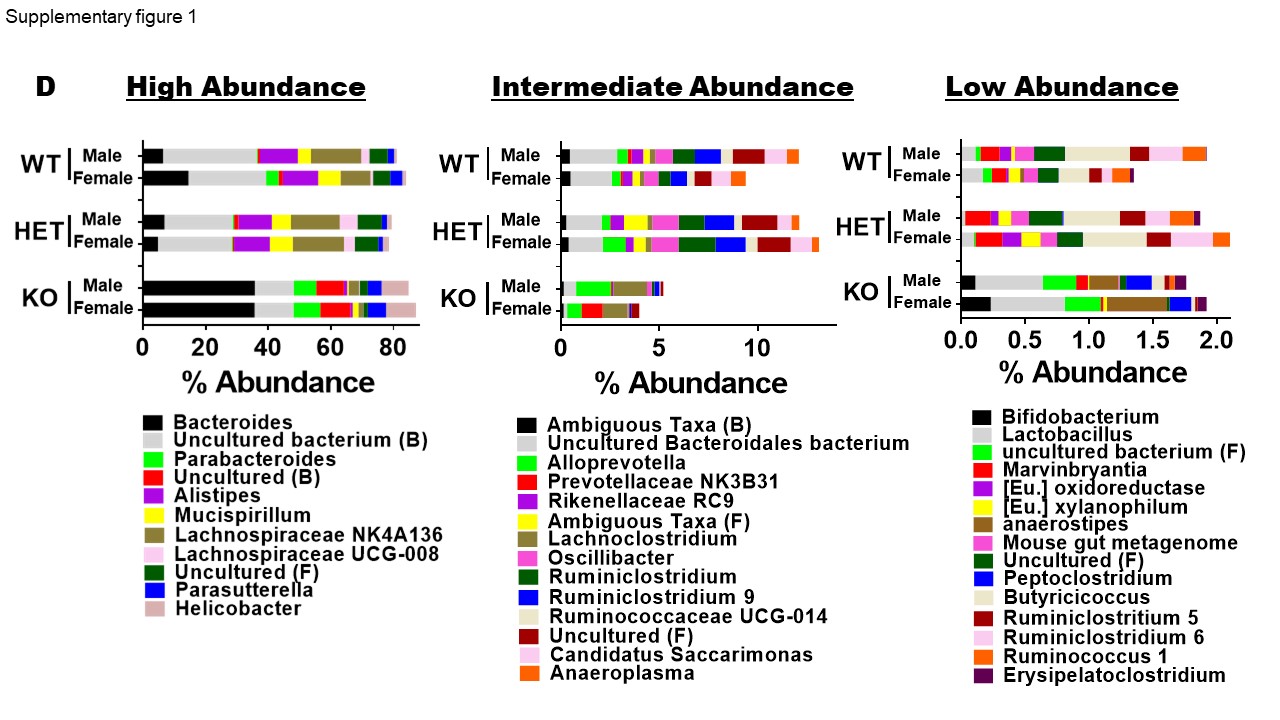

Supplement: FIGURE S1 — VIP deficiency causes phylogenetic lineage alterations within fecal gut microbiota. Horizontal stacked-bar graphs for (A) phyla, (B) class, (C) order, and (D) genus taxonomic levels. Color represent means of % abundance levels corresponding to the taxonomic name for that same color associated with each graph. Graphs are organized into high-, medium, and low % abundance categorized by genotype and sex. Forty of the 59 genera are graphed. [file Data_Sheet_1.zip › Image 1D.JPEG]

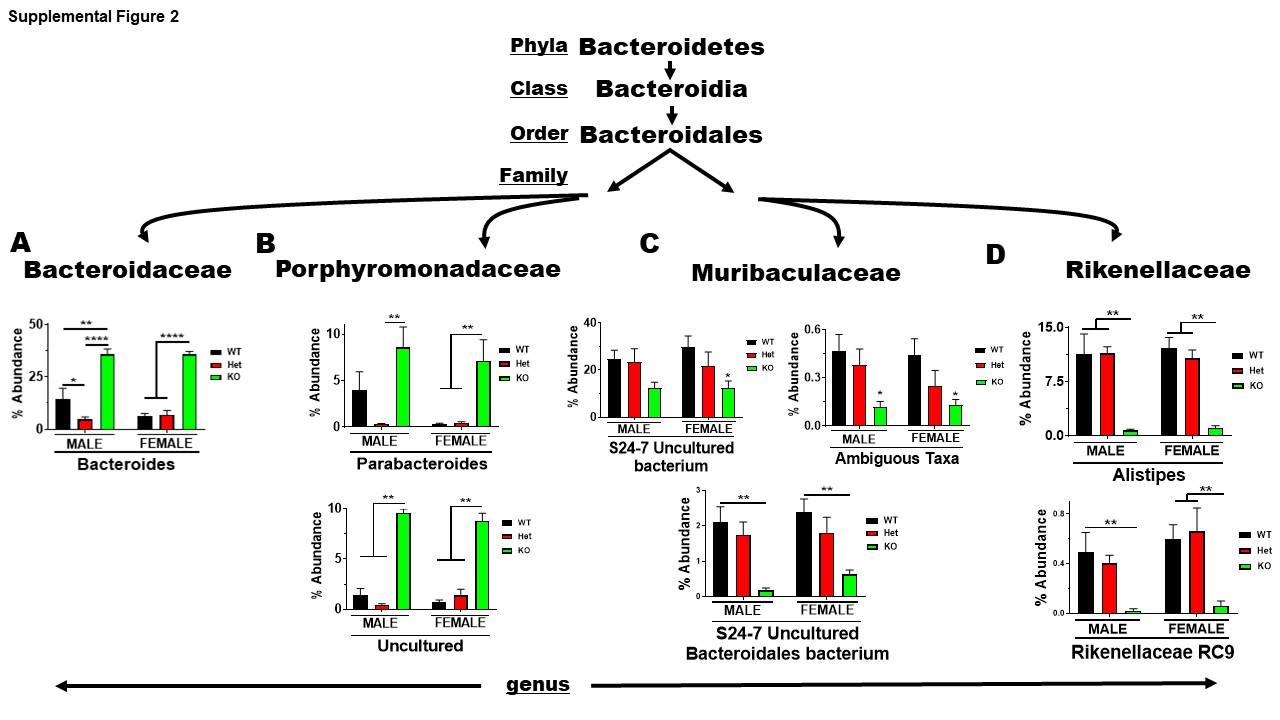

Supplement: FIGURE S1 — VIP deficiency causes phylogenetic lineage alterations within fecal gut microbiota. Horizontal stacked-bar graphs for (A) phyla, (B) class, (C) order, and (D) genus taxonomic levels. Color represent means of % abundance levels corresponding to the taxonomic name for that same color associated with each graph. Graphs are organized into high-, medium, and low % abundance categorized by genotype and sex. Forty of the 59 genera are graphed. [file Data_Sheet_1.zip › Image 2.JPEG]

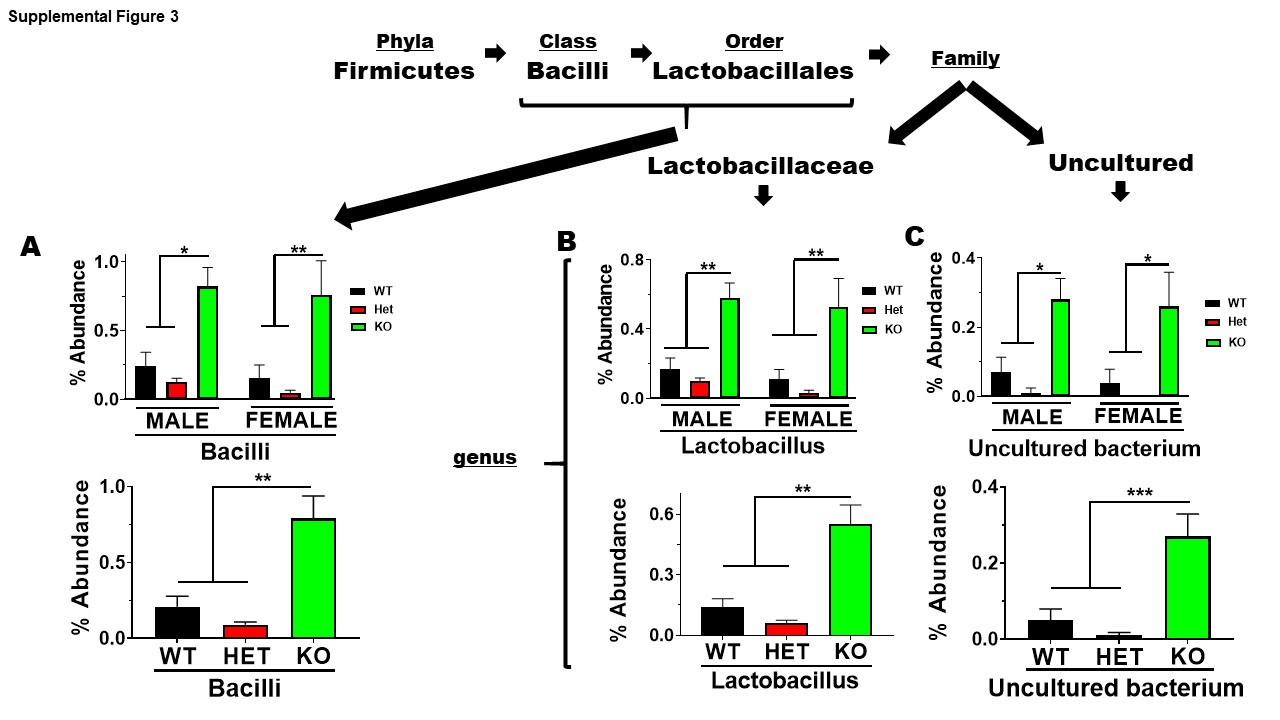

Supplement: FIGURE S1 — VIP deficiency causes phylogenetic lineage alterations within fecal gut microbiota. Horizontal stacked-bar graphs for (A) phyla, (B) class, (C) order, and (D) genus taxonomic levels. Color represent means of % abundance levels corresponding to the taxonomic name for that same color associated with each graph. Graphs are organized into high-, medium, and low % abundance categorized by genotype and sex. Forty of the 59 genera are graphed. [file Data_Sheet_1.zip › Image 3.JPEG]

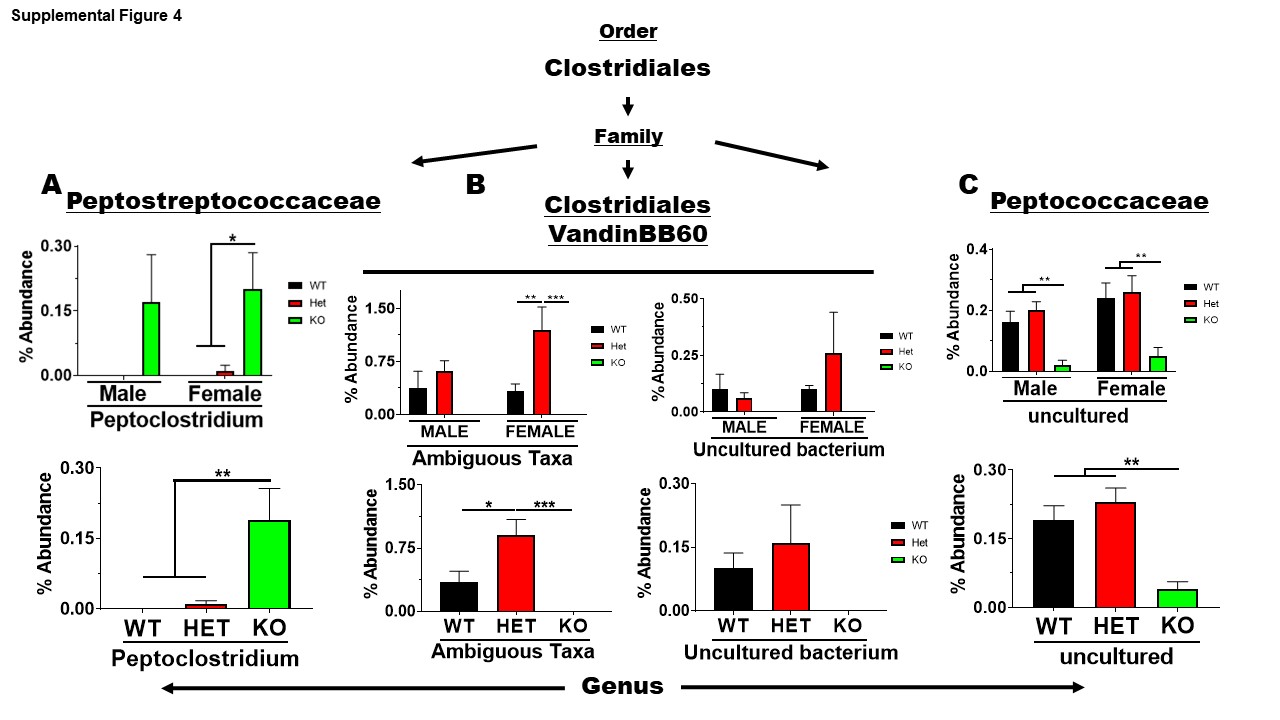

Supplement: FIGURE S1 — VIP deficiency causes phylogenetic lineage alterations within fecal gut microbiota. Horizontal stacked-bar graphs for (A) phyla, (B) class, (C) order, and (D) genus taxonomic levels. Color represent means of % abundance levels corresponding to the taxonomic name for that same color associated with each graph. Graphs are organized into high-, medium, and low % abundance categorized by genotype and sex. Forty of the 59 genera are graphed. [file Data_Sheet_1.zip › Image 4.JPEG]

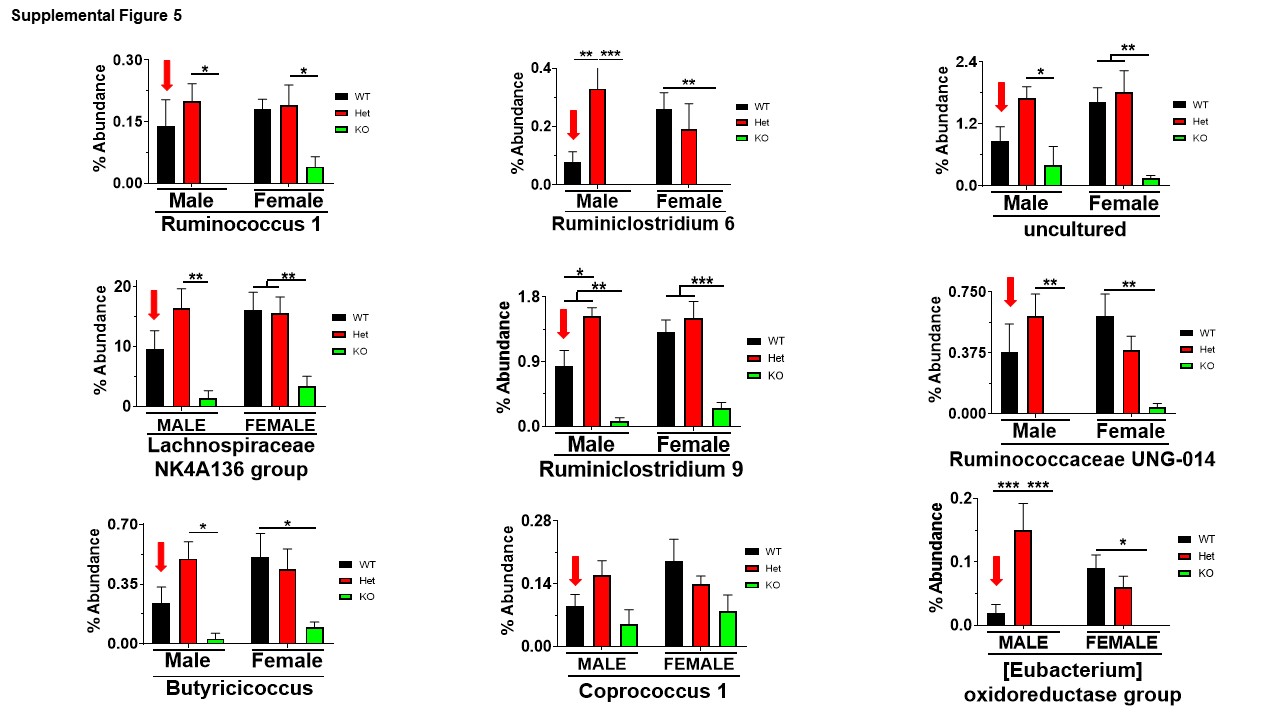

Supplement: FIGURE S1 — VIP deficiency causes phylogenetic lineage alterations within fecal gut microbiota. Horizontal stacked-bar graphs for (A) phyla, (B) class, (C) order, and (D) genus taxonomic levels. Color represent means of % abundance levels corresponding to the taxonomic name for that same color associated with each graph. Graphs are organized into high-, medium, and low % abundance categorized by genotype and sex. Forty of the 59 genera are graphed. [file Data_Sheet_1.zip › Image 5.JPEG]

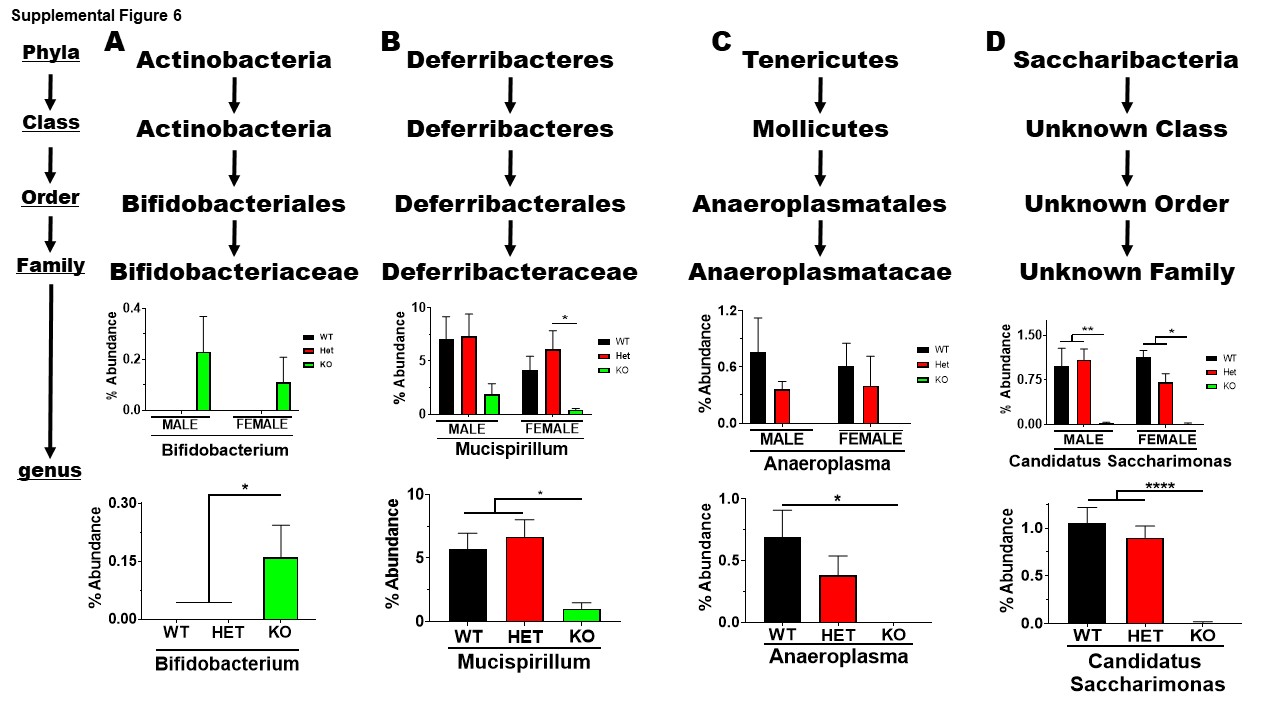

Supplement: FIGURE S1 — VIP deficiency causes phylogenetic lineage alterations within fecal gut microbiota. Horizontal stacked-bar graphs for (A) phyla, (B) class, (C) order, and (D) genus taxonomic levels. Color represent means of % abundance levels corresponding to the taxonomic name for that same color associated with each graph. Graphs are organized into high-, medium, and low % abundance categorized by genotype and sex. Forty of the 59 genera are graphed. [file Data_Sheet_1.zip › Image 6.JPEG]
